# Minimal Sensor Configuration for Human Activity Recognition in Patients With Hip Osteoarthritis: Proof-of-Concept Study

**DOI:** 10.2196/95116

**Published:** 2026-09-16

**Authors:** Noor Alalem, Valérie Duay, Angelo Di Benedetto, Kevin Rose-Dulcina, Didier Hannouche, Stéphane Armand, Xavier Gasparutto

**Affiliations:** 1 Kinesiology Laboratory, Geneva University Hospitals and University of Geneva, Rue michel servet 1, Geneva, Geneva, Switzerland, 41 782571877; 2 Centre of Research on Skeletal Muscle and Movement, University of Geneva and Geneva University Hospitals, Geneva, Switzerland; 3 HEPIA - HES-SO University of Applied Sciences and Arts Western Switzerland, Geneva, Geneva, Switzerland; 4 Division of Orthopaedics and Traumatology, Geneva University Hospitals, Faculty of Medicine, Geneva, Geneva, Switzerland

**Keywords:** activities of daily living, ADL, gated recurrent unit, GRU, human activity recognition, HAR, hip osteoarthritis, OA, inertial measurement unit, IMU, neural networks, total hip arthroplasty, THA

## Abstract

**Background:**

Hip osteoarthritis (OA) impairs function during activities of daily living (ADL); however, objective functional assessment methods are lacking. Objectively measuring and characterizing functional deficits during ADL can help rehabilitation specialists target the activities in which patients experience the greatest difficulties. To characterize daily living function, ADL must first be accurately identified. Human activity recognition (HAR) models based on wearable sensor measurements, such as inertial measurement units (IMUs), have been implemented in individuals with various functional impairments but not yet in people with end-stage hip OA. In addition, the balance between HAR accuracy and wearability, which is crucial for patients’ acceptance, remains unclear. Finally, to assess deficits of pathological populations, comparison with asymptomatic controls is necessary.

**Objective:**

This study aimed to evaluate the minimal accurate IMU configuration for HAR, from 8 to 1 IMU, in patients with hip OA and asymptomatic controls for mobility-related activities.

**Methods:**

Data from 20 patients and 9 controls were included. Participants completed a tour of ADL in the hospital vicinity while equipped with 8 IMUs. Activities included gait on flat ground, gait up and down a ramp, stair ascent/descent, turns, sitting up/down, and static sitting. Bidirectional gated recurrent unit models were trained to classify ADL based on 3D accelerations and angular velocities of multiple IMU configurations. Model accuracy was assessed on test sets of patient and control data using the Cohen Kappa( κ).

**Results:**

The resulting κ values were 0.95 for 8 IMUs, 0.93 for 4 IMUs, 0.90 for 2 IMUs, and 0.79 for 1 IMU. The accuracy was higher for controls than for patients. The best 2-IMU configurations were 2 shanks and 2 feet, and the best single-IMU configuration was the shank.

**Conclusions:**

Reducing the number of IMUs from 8 to 2 showed only a minimal decrease in κ, suggesting that minimal IMU setups could be as accurate as larger setups. Regarding gait detection, a single IMU was sufficient to reach very high accuracy (κ≥0.90). The single pelvic IMU showed the lowest accuracy for gait while still reaching high accuracy (κ>0.65). These results underline the potential of single-IMU or 2-IMU configurations to recognize mobility activities under semistandardized conditions. However, given the small sample size, these findings should be validated in larger and more heterogeneous hip OA cohorts before any clinical application can be considered.

## Introduction

Osteoarthritis (OA) is one of the leading causes of disability [1]. Individuals with end-stage hip OA experience joint pain, swelling, stiffness, and functional limitations [2]. This hinders their ability to perform activities of daily living (ADL), such as walking, ascending or descending stairs, transitioning from a chair, and performing household chores. The extent of functional limitations in people with hip OA depends on the amount of structural disease progression and associated symptoms [2]. There is no cure for hip OA; however, its common treatment is total hip arthroplasty (THA), which aims to alleviate pain and restore function [3]. However, it is recognized that undergoing THA does not imply reversal of disability and complete restoration of function [2], and that patients do not reach the functional level of healthy controls [4]. The management of hip OA hence requires an understanding of the symptoms and functional limitations of the individual [5], which in turn requires evaluation of the influence of hip OA on ADL characteristics. Assessing the amount of ADL performed by patients as well as the differences in activity quantity before and after THA could provide information about their functional levels and their ability to perform a certain activity. Moreover, measuring functional deficits during ADL upon recognizing activity type can help identify activities during which patients face the greatest limitations, allowing rehabilitation practitioners to target such activities for patient-specific functional improvement [6]. Ultimately, hip OA is characterized by mobility impairments that directly affect how individuals perform everyday activities. Therefore, monitoring ADL related to real-world mobility, such as crossing the street, cooking, dressing, or cleaning, could be clinically relevant, as they are related to pain, stiffness, societal participation restrictions, and quality of life.

To assess the functional status of patients with hip OA, clinicians mostly rely on patient-reported outcome measures [7] or objective standardized tests (eg, 6-min walk test, 30 s chair-stand test, etc) [8]. The former tools are subjective [9] and assess a patient’s perception of their ability to perform a functional task and were reported as more influenced by pain than objective function [10]. The latter tests measure capacity—that is, what individuals “can do” in supervised settings—rather than performance, which reflects function in ADL or, as defined by the World Health Organization, what individuals “actually do” in their natural environment [11]. Therefore, objective tools that can measure patients’ functional performance in daily living settings are needed to fully capture patients’ function.

Wearable inertial measurement units (IMUs) can capture unsupervised movement data, which can then be processed into clinically interpretable information [12]. Thus, IMUs have the potential to (1) provide mobility outcomes measured during ADL that could support clinical trials and (2) improve the understanding of patient function [13]. Nevertheless, while IMUs can collect large amounts of data over extended periods, the resulting datasets can quickly become tremendous, complex to treat, and most importantly, clinically uninterpretable [14]. Thus, the first step after data collection is to identify the activities performed by the patients, based on the available IMU signals, that is, angular velocities and linear accelerations, before being able to characterize them.

Human activity recognition (HAR)—a popular approach that uses machine learning (ML) algorithms for automatic activity classification—has been successfully used to identify ADL from IMU data. Most existing HAR models rely on traditional ML methods such as Random Forests, Decision Trees, and Support Vector Machines. Although they achieved good performance, they relied on handcrafted feature extraction, which can only capture shallow features from the input data [15]. In recent years, deep learning neural networks, including convolutional neural networks (CNNs), recurrent neural networks, long short-term memory networks, and gated recurrent units (GRUs), have become popular [16]. GRUs were designed to reduce computational complexity associated with long short-term memory networks [17] and have shown advantages in modeling temporal sequences, outperforming existing ML methods in classification accuracy [13,14]. Since GRUs can automatically extract features from raw time-series data, without any human intervention, they make it possible to identify unknown movement patterns [18]. In addition, hybrid networks such as CNN-bidirectional GRU (Bi-GRU) and transformer-based attention models have been recently reported as potential solutions for HAR [17,18].

Several studies have developed deep learning–based HAR models to recognize mobility-related activity in healthy participants using multi-IMU setups [17,19]. The most common classified activities include walking, ascending and descending stairs, standing, sitting, and lying [16]. The most reported metric was accuracy, ranging from 0.90 to 0.97 [17,19,20]. One study built a Bi-GRU network, which obtained accuracies of 0.95, 0.95, and 0.99 when tested on 3 different datasets with healthy participants [21]. Bi-GRUs were selected in this study as a balanced trade-off between temporal modeling, making them well suited for HAR and relatively small datasets [22]. Moreover, Bi-GRUs were used in many hybrid HAR architectures such as CNN-BiGRU as they improved model performance [22-24]. Transformer-based attention models and activity graph-based CNNs have also been recently reported as potential solutions for HAR [17,25].

A large range of sensor configurations was used in the literature for HAR [16]. However, one perspective of this study is using the HAR model for classifying gait and measuring its spatiotemporal parameters (ie, walking speed, stride length, etc), in addition to hip range of motion in patients with hip OA since they are outcomes of clinical interest [26]. The configurations of previous studies [27] to assess gait and mobility parameters included (1) 7 IMUs: waist, thighs, shanks, and feet; (2) 4 IMUs: thighs and shanks; (3) 3 IMUs: waist and feet; (4) 2 IMUs: waist and thigh; (5) 2 IMUs: waist and shank; (6) 2 IMUs: waist and foot; (7) 2 IMUs: shanks; (8) 2 IMUs: feet; (9) 1 IMU: waist; (10) 1 IMU: thigh; (11) 1 IMU: shank; (12) 1 IMU: foot; (13) 1 IMU: chest; and (14) 1 IMU: wrist. The shanks configuration was the most used among multisensor configurations [28-30], followed by the shanks and thighs configuration [31-33]. Regarding single-sensor configurations, the waist was the most reported [33-35], followed by the foot [36-39]. Regarding populations, most models were trained using healthy participant data [16], and other models were trained using data from patients with knee OA [31] as well as those with Parkinson disease, stroke, or older adults who are fall-prone or frail [15,16].

The existing HAR models have 4 main limitations. First, movement characteristics and compensations of patients with hip OA are different from those of healthy people or people with knee OA [21,40]. Indeed, it was shown that HAR models trained on healthy participants only could not accurately classify patient activities [35]. Still, since comparison with controls is required to assess patients’ limitations, an ideal HAR model would be trained and evaluated with both patients with hip OA and controls. Second, the optimal number and configuration of IMUs for accurate activity recognition in patients with hip OA have not yet been systematically investigated. Identifying the minimal IMU configuration with acceptable recognition would greatly help improve patient acceptance [36] and the subsequent possibility of following cohorts. The third limitation in this context was the common class imbalance issue in datasets, that is, there is a large discrepancy between the number of observations per class [16]. This is largely because walking is the most frequent daily living activity compared to less frequent activities such as taking stairs or turning. Consequently, models trained on unbalanced datasets may appear accurate simply by correctly predicting the most prevalent class, giving a misleading impression of the overall performance [37]. When compared to accuracy, precision, recall, and *F*_1_ score metrics, the Cohen κ was reported to be more robust and tolerant to variations in class distribution [38] and could be an appropriate metric to evaluate model performance on imbalanced datasets. The fourth limitation is the potential misplacement of IMUs by patients in daily life. To the best of our knowledge, no prior study has addressed the variability of IMU positions during daily life, despite this being an inevitable factor.

Given the limitations of the literature, the aim of this study was (1) to evaluate the performance of HAR models in patients with hip OA and controls as a function of IMU number and configuration and (2) to compare accuracy between patients with hip OA and controls.

## Methods

### Ethical Considerations

This observational study was conducted in accordance with the principles of the Declaration of Helsinki. Ethical approval was obtained from the Comité Cantonal d’Éthique de la Recherche (CCER-2017‐00817, Geneva, Switzerland). Written informed consent was obtained from all participants prior to their inclusion in the study. Data, including videos of participants, were stored according to the Swiss Human Research Ordinance Art. 5; the videos did not include participants’ faces. For data protection purposes, the data were stored on a local and secure server accessible only to the clinicians and researchers involved in this project with due rights from the ethics committee of Geneva (Comité Cantonal d’Éthique de la Recherche). Participants consented to the use of their data for research purposes, given that proper anonymization and protection measures would be taken.

### Recruitment

This study included 21 patients with end-stage hip OA and 10 asymptomatic controls. The inclusion criteria for patients were (1) aged 30 to 85 years, (2) planned for an elective primary THA due to OA, (3) anterior/posterior surgical approach, and (4) able to walk 10 m without assistance. The inclusion criteria for controls were (1) matched for age, (2) no neuromusculoskeletal disorders that could affect mobility, and (3) able to walk 10 m without assistance. Sex distribution was tested using the chi-square test, and differences in age, height, and BMI were evaluated using nonparametric Wilcoxon *t* tests (*P*<.05).

### Protocol

Participants were asked to perform a 10-minute semistandardized tour in the hospital vicinity, with activities including walking on flat ground, walking up and down a ramp, ascending and descending stairs, turning, sitting up/down, and static sitting. They were equipped with 8 IMUs (Physilog 6, MindMaze, Switzerland) placed on the thorax, pelvis, thighs, shanks, and feet, and simultaneously video-recorded with a smartphone to provide ground truth for activity identification. Participants wore their usual clothes during the tour and sandals equipped with pressure insoles (not used in the current study).

**Figure 1.**
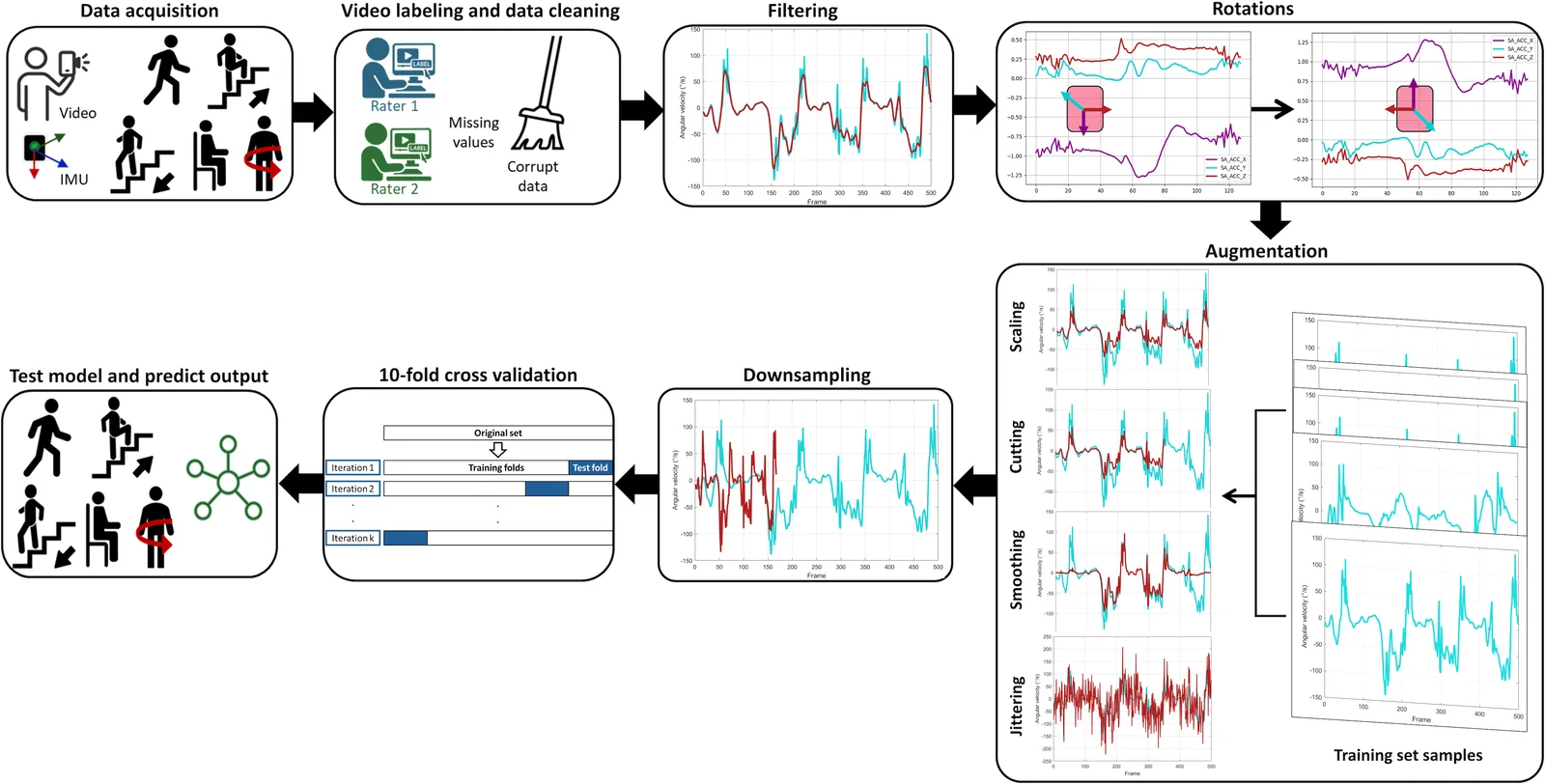
Human activity recognition workflow, including data acquisition, preprocessing, data augmentation, model training, and activity classification steps. IMU: inertial measurement unit.

The dataset consisted of 3-dimensional linear acceleration and angular velocity signals acquired at 128 Hz, along with activity labels assigned to each timestamp in videos with BORIS (version 8.27.10) [39] by a single operator. The interoperator agreement of task labeling was evaluated using a second operator on a subset of 7 participants. The general workflow of the study presented in Figure 1 describes the workflow of the HAR algorithm and will be detailed in the following sections.

### Preprocessing

IMU recordings containing corrupted data (ie, IMU malfunction) were removed.

Brief transitions between activities (ie, sit-to-stand, sit-down transitions, and 90° turns) were also excluded to simplify classification since these transitions represent short intertask movements rather than discrete activities and could confuse the model. Moreover, walking on flat ground and up and down a ramp (5° slope) were grouped as “Gait,” and left and right 180° turns (U-turns) were grouped as “Turns” for further simplification. As a result, 5 activities were selected as outputs: gait, stair climbing, stair descent, turns, and static sitting. All IMU signals were filtered using a Butterworth low-pass filter (zero-phase, cutoff: 2 Hz, second order).

### Data Augmentation

To simulate IMU placement variability in addition to data augmentation, rotations were applied to the IMU coordinate systems. Rotations included 2 random angles, three 180° flips (1 per axis), and 1 inversion for all axes per segment. The assumption was that rotating the acceleration and angular velocity signals could make the model more robust to random sensor placements in daily life. Besides rotation, 4 data augmentation methods commonly used in image processing were used to handle class imbalance [41]: (1) scaling, (2) jittering, (3) cutting, and (4) smoothing [42]. The 4 methods were parametrized and randomly applied to the data to synthetically generate observations for the less frequent classes, to match the number of occurrences of gait, the most frequent one. More precisely, scaling was implemented by multiplying each sensor signal by a random amplitude factor proportional to its norm. Jittering was based on adding Gaussian noise to the signal amplitude, where noise magnitude was 1% of the signal’s norm. Cutting was implemented using a cut ratio, determined randomly, while enforcing the preservation of a minimum of 100 samples to avoid overly short signals. Smoothing relied on a Hann-window convolution filter to smooth signal edges. While rotations were applied equally to all classes to simulate IMU placement variability, scaling, jittering, cutting, and smoothing were applied exclusively to minority classes to match the observation count of gait, the majority class. This asymmetric strategy was intentional, as it directly addresses class imbalance while avoiding unnecessary inflation of the majority class data. The class-balanced weighted cross-entropy loss function further compensated for any remaining imbalance during training.

Before augmentation, the dataset was randomly split into training and test sets with a 75:25 ratio, leaving 7 participants (3 controls and 4 patients) for the test set. The augmentation process was only applied to the training set, leaving the test set originally imbalanced. Before augmentation, gait was the most frequent activity, accounting for 60% (46,080/76,800 frames) of the total tour’s duration (~6 min). After applying rotations, all activities, including gait, were augmented, resulting in the same uneven distribution; gait still accounted for 60% (276,480/460,800 frames) but increased to approximately 36 minutes and 276,480 frames. After augmenting the other classes to match gait count with the 4 mentioned methods, classes were evenly distributed, each representing 20% (276,480/1,382,400 frames) of the total duration with the same count as gait (~36 min).

### Downsampling

All IMU signals were scaled using the StandardScaler library in Python 3.12 and downsampled by a factor of 3 (43 Hz) [43,44] to reduce the computation time caused by augmentation.

### Bi-GRU Architecture

**Figure 2.**
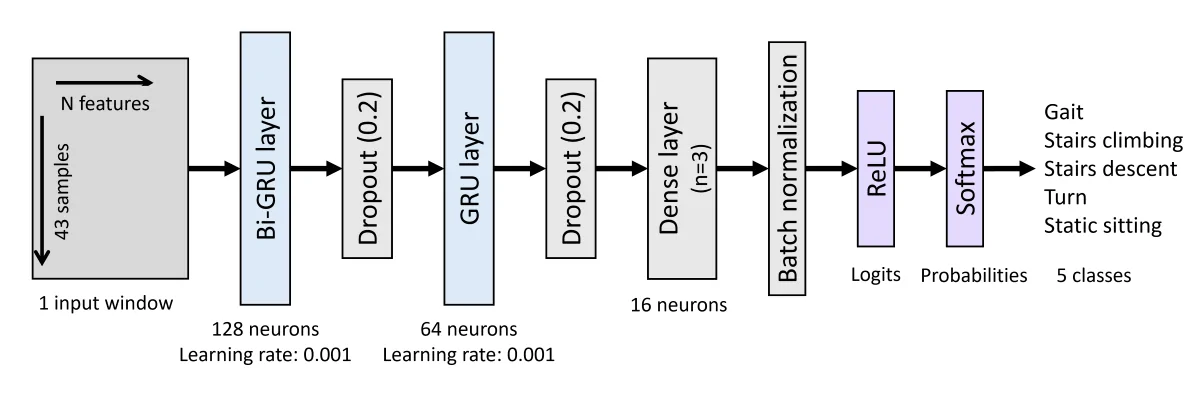
Architecture of the proposed bidirectional gated recurrent unit (Bi-GRU) model illustrating the input data, network layers, and training hyperparameters. GRU: gated recurrent unit; ReLU: rectified linear unit.

The proposed network structure of the Bi-GRU model is presented in Figure 2. To maintain the time dependency of the data points in each activity, a sliding window (1-s length) with an overlap of 300 milliseconds was used to input the 3D accelerations and angular velocities to the GRU model, and output windows were the 5 activities. The input windows were fed into a Bi-GRU with 128 neurons, followed by a unidirectional GRU with 64 neurons to extract temporal features [16]. After each GRU layer, a dropout layer was added to reduce overfitting. Finally, the output of the network was obtained from 3 dense layers (fully connected) and a rectified linear unit function. In addition, a batch normalization layer was added after the dense layers to accelerate the training process [45]. The model was trained with a learning rate of 0.001. The final output probabilities of each class were obtained using the softmax function. The output classes were further classified into an “Unknown” class if the Softmax probability fell below a rejection threshold, defined as Q1=−1.5 × IQR of the distribution of maximum softmax probabilities derived from correctly classified known-class samples in the validation set [46]. The training and prediction processes were run using a 13th Gen Intel Core i9-13900 at 2 GHz (Intel Corp). The training and testing were performed once for each IMU configuration, yielding a unique model per configuration. Every model was trained using a class-balanced weighted cross-entropy loss function to account for class imbalance. The network parameters were optimized with the Adam optimizer, with a consistent batch size of 512 [47]. The computational time required for training each model configuration was approximately 30 minutes.

### IMU Configurations

**Figure 3.**
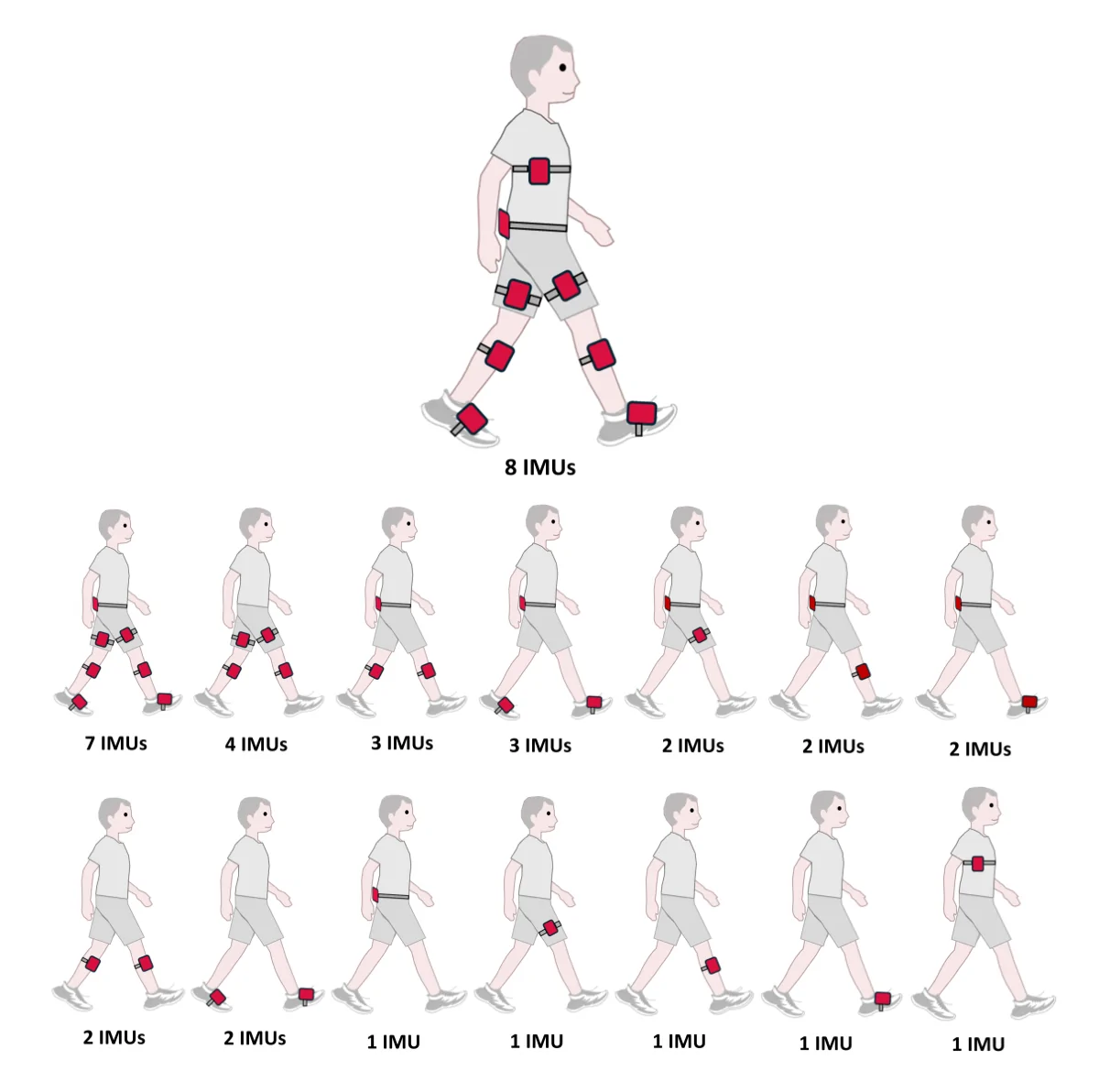
Inertial measurement unit (IMU) configurations evaluated for human activity recognition, showing the sensor placements for each setup, ranging from 8 to 1 sensor.

We tested the configurations mentioned in the literature in addition to 3 other configurations (Figure 3): (1) 8 IMUs: trunk, pelvis, thighs, shanks, and feet, (2) 3 IMUs: pelvis and shanks. The wrist IMU was not used in this study as it was shown to yield higher variability and lower correlation when estimating temporal gait parameters [48], as opposed to the chest and thigh locations [41,42]. For configurations using one side of the limbs (eg, waist and thigh, waist and shank, thigh, shank, etc), the operated hip side was chosen for patients, and an arbitrary side for controls.

### Model Performance

For each configuration, a 10-fold group cross-validation was performed on the training set (n=22 participants, 75% of the dataset). To avoid data leakage, each participant was used in the validation set only once. This group cross-validation approach was chosen as it provides subject-independent performance estimates, as recommended for HAR studies and ML models developed with limited sample sizes [45,46]. The outcome of the training was the average *F*_1_ score across all folds, and the best model among the 10 was selected based on the highest *F*_1_ score. The number of epochs was set to 70, and to prevent overfitting, early stopping was applied when the validation *F*_1_ score stopped increasing (Figure S1, Multimedia Appendix 1). To ensure reproducibility, random seeds were set to 42. The best model was used to predict the output classes on the unseen test set, and the resulting overall precision (1), recall (2), specificity (3), and *F*_1_ score (4) were reported. In addition, to check for the ability of the model to recognize each activity individually, per-class metrics were calculated. The data processing, model architecture, and training were developed and implemented using Python 3.12.0 with the libraries NumPy, Pandas, Torch, and Scikit learn.


(1) $$Precision=\frac{\text{TP}}{\text{TP}+\text{FP}}$$

(2) $$Recall=\frac{\text{TP}}{\text{TP}+\text{FN}}$$

(3) $$Specificity=\frac{\text{TN}}{\text{TN}+\text{FP}}$$

(4) $$F_{1}\text{score}=\frac{2\cdot (\text{precision}\cdot \text{recall})}{\text{precision}+\text{recall}}$$

To assess the overall agreement between the model and the operators (ie, the model’s predictions vs the labels of operator 1), the Cohen κ statistic—a measure of agreement between categorical variables—was used. A κ of 1 indicates perfect agreement between raters, while a κ of 0 indicates agreement due to random chance [49]. κ was calculated over output windows of 1 second (43 frames).

Cohen κ values were interpreted as follows: 0 to 0.20, very low agreement; 0.21 to 0.40, low agreement; 0.41 to 0.60, medium agreement; 0.61 to 0.80, high agreement; 0.81 to 1.0, very high agreement [50]. 95% CIs were also calculated for the κ values to estimate the true κ range and assess clinical acceptability [50].

### Interoperator Agreement

A second operator additionally labeled videos of the test set (n=7 participants). Activities were labeled at each video frame and then segmented into 1-second windows with an overlap of 300 milliseconds. The interoperator agreement was then evaluated with the Cohen κ.

## Results

### Participants

After the exclusion of 2 participants due to unexpected signal loss, 20 patients with hip OA and 9 asymptomatic controls (Table 1) were included in this study’s analysis.

**Table 1.** Participant characteristics.

Characteristics	Patients (n=20)	Controls (n=9)	*P* value
Sex (female), n	11	7	.08
Age (y), mean (SD)	60.7 (6.9)	69 (7.9)	.29
Weight (kg), mean (SD)	72.3 (13.2)	64.1 (13.3)	.04
Height (cm), mean (SD)	171.2 (11.4)	163 (10.1)	.06
BMI (kg/m^2^), mean (SD)	24.8 (3.0)	23.41 (3.1)	.48

### Kappa vs IMU Setup

**Figure 4.**
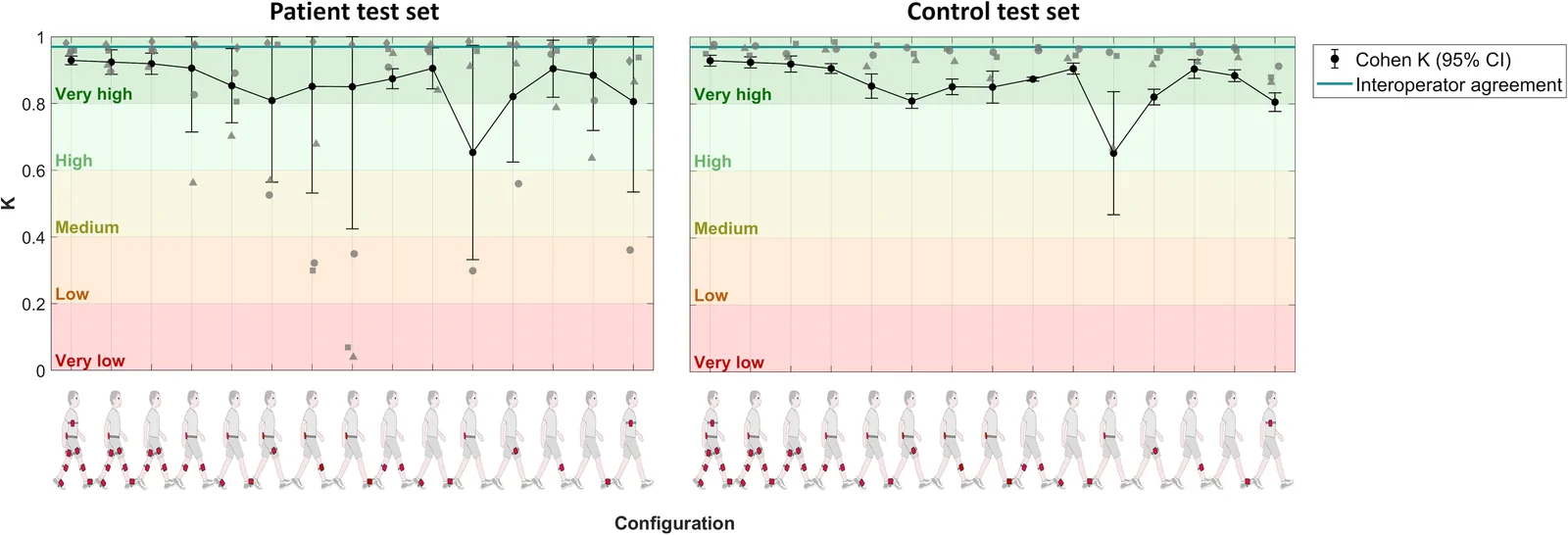
Cohen κ values with 95% CIs across inertial measurement unit (IMU) configurations for the patient (n=4) and control (n=3) test sets.

The 8-IMU configuration obtained the highest agreement (κ=0.95) with a very small CI for the patient test set (Figure 4). Decreasing the number of IMUs led to lower κ values and larger CIs, indicating lower accuracy and higher variability. Configurations with the lowest agreement and highest variability included the pelvis (eg, 2-IMU pelvis-thigh, 2-IMU pelvis-shank, 2-IMU pelvis-foot, and 1-IMU pelvis). However, the configurations with only 2 IMUs on the shanks or feet had very high agreement accuracy and low variability. Single-IMU configurations varied from low to high agreement with increased CIs. The shank demonstrated the highest κ with the lowest variability among single-IMU configurations.

### Kappa vs Activity

**Figure 5.**
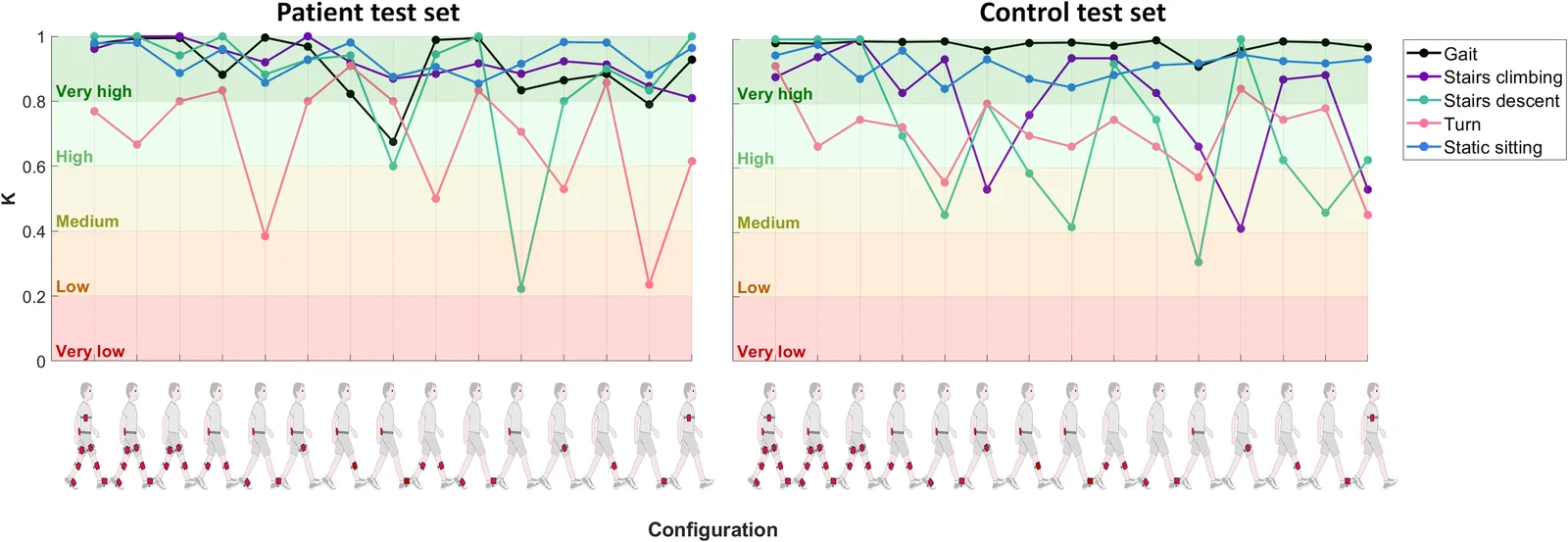
Per-class Cohen κ values across inertial measurement unit configurations for each activity in the patient (n=4) and control (n=3) test sets.

Most configurations allowed activity recognition in patients with end-stage hip OA with sufficient levels of agreement (κ>0.6). The accuracy of task detection was dependent on the task itself (Figure 5). Indeed, gait and static sitting presented very good results (κ>0.8) for all configurations, while turns highly depended on the configuration, with agreements ranging from low to very high. Stair ascent and descent were also well detected in most configurations (κ>0.6). Regarding the “Unknown” class, the frequency of its prediction ranged from 2.3% to 12.8% depending on IMU number and configuration, and model performance remained constant as long as the threshold was less than 0.8.

### Patients vs Controls

Regarding asymptomatic controls, recognition was accurate in all configurations (κ>0.80) with small CIs and was sufficiently accurate in the pelvic configuration, despite its lower κ of 0.65. The quality of recognition varied from task to another, as also observed with patients. Gait and static sitting showed consistently very high agreement in all configurations, while turns, stair ascent and descent showed moderate recognition quality (medium agreement) in 3 (20%) of the configurations.

Precision, recall, *F*_1_ score, and specificity metrics for patients and controls are presented in Multimedia Appendix 1 (Figures S3 and S4).

## Discussion

This study was the first to (1) develop an HAR model trained using data from patients with end-stage hip OA and (2) perform a thorough comparison of commonly used single- and multi-IMU configurations.

### Principal Findings

This study’s 8-IMU, 7-IMU, 4-IMU (thighs and shanks), 3-IMU (pelvis-feet), and 2-IMU (shanks and feet) models achieved very high levels of agreement with small CIs in patients with hip OA and asymptomatic controls. The good accuracy of shanks to identify mobility-related activities aligns with previous research highlighting the most accurate IMU configurations for measuring spatiotemporal gait parameters in children with cerebral palsy [51]. Interestingly, the differences in accuracy between the 8-IMU, 4-IMU, and best-performing 2-IMU configurations (ie, shanks, feet) were minimal for patients with hip OA, where the shanks and feet obtained precision, recall, specificity, and *F*_1_ score values >0.9 as shown in Figures S3 and S4, Multimedia Appendix 1. The agreement remained very high, with κ values between 0.85 and 0.95, suggesting that 2 well-placed IMUs can provide a similar accuracy level as those of a full-body IMU setup. Single-IMU configurations showed lower accuracy, with 2 configurations achieving high agreement (0.78 and 0.75 for the shank and trunk), and 3 configurations obtaining medium agreement (0.59, 0.50, and 0.45 for the thigh, pelvis, and foot). Despite being lower than those of multi-IMU configurations, these outcomes may be acceptable when assessing ADL, especially in studies focusing on gait. Indeed, all single-IMU setups achieved high to very high agreement in gait detection. Moreover, a κ>0.60 was reported to be equivalent to an agreement of 90% [50], which suggests very good classification performance for the single-IMU shank and trunk configurations. For 2-IMU configurations that combined the pelvis with the thigh, shank, or foot, the models generally performed worse—exhibiting higher variability and lower accuracy—compared to configurations that did not include the pelvic IMU. Similarly, the pelvic IMU alone showed lower accuracy compared to other single-IMU configurations. This may be explained by the lower amplitudes of acceleration and angular velocity that the pelvic IMU measures, resulting in a lower signal-to-noise ratio that could yield an undesired noisy signal. Moreover, since most of the activities classified were ambulatory (ie, gait, stairs, and turns), they were more accurately reflected by thigh, shank, or foot IMUs than by the pelvis given the swing movements occurring in these segments. Thus, the pelvic IMU might have confused the model rather than contributed to useful information in the 2-IMU configurations.

Patients showed lower κ values and greater variability across configurations when compared with asymptomatic controls. However, recognition accuracy still depended on activity type in both groups, where gait and static sitting were the most robust, while stairs and turns showed lower accuracy and higher variability. Stair ascent and descent were often confused with gait due to similar cyclic movement patterns. Similarly, turn recognition showed lower accuracy due to its misclassification as gait, as turning includes multiple steps, which makes it closer to gait. Additionally, variability in turning strategies (eg, pivot turns vs multistep turns) may have contributed to misclassification. The discrepancy between precision and recall observed in patients (Figure S3, Multimedia Appendix 1) was not observed in controls’ metrics (Figure S4, Multimedia Appendix 1), where precision and recall were consistently high (≥0.95) in all configurations, and slightly lower in the pelvic IMU configuration (recall=0.90, precision=0.93). These results suggest that HAR in control participants was more accurate, and its high precision and recall encourage the applicability of several model configurations for assessing activity quantity and quality in healthy individuals.

The narrow CIs observed for most configurations, particularly for the 8-IMU, 4-IMU, and 2-IMU shanks/feet setups, suggest stable and consistent model performance despite the limited sample size. Moreover, the κ values were obtained on a fully unseen test set, providing an unbiased estimate of model performance. However, the estimate should still be interpreted with caution due to the small sample size. While the numerical difference in κ between the 8-IMU, 4-IMU, and best-performing 2-IMU configurations appears small, it is important to note that performance degradation remains present and variability increased with fewer IMUs. The clinical acceptability of these configurations will ultimately depend on the intended application as discussed in the “Selecting the Optimal Model” section.

### Comparison With Prior Work

Various studies have developed HAR models and achieved high accuracy, but most models were trained using data from healthy populations [17,30], which are not necessarily applicable to patients with pathologies of the locomotor system [35]. Two studies have previously explored multiple IMU configurations to classify activity in healthy participants. Rahn et al [52] developed a CNN-based HAR model and investigated several smartphone-IMU configurations with 5 IMUs placed on the upper arm, wrist, lower back, upper thigh, and ankle and a smartphone placed in the front right pocket. They reported *F*_1_ scores between 0.65 and 0.87 for 1 IMU configurations, which are lower than the *F*_1_ scores achieved in this study’s single-IMU setups (>0.90 for the thigh, shank, and trunk). As for their 2-sensor setups (1 IMU and 1 smartphone), they reported *F*_1_ scores between 0.68 and 0.97, which are comparable to this study’s 2-IMU setups (0.75 to 0.98). In a similar study, Dalton and O’Laighin [53] explored multiple IMU configurations positioned on the chest, waist, wrist, and ankle with very high agreement (κ ranging from 0.77 to 0.95). These κ values were higher than those reported in the present study for the same configurations (1-IMU foot and 1-IMU pelvis: 0.45 and 0.50). This may be explained by the fact that our Bi-GRU model was trained with more data from patients with hip OA than from healthy controls.

### Selecting the Optimal Model

The criteria for selecting the “best” HAR model rely on its future application, that is, whether the aim is to assess activity quantity or quality. To evaluate activity quantity (ie, how much a patient performs), both false positives and false negatives should ideally be 0 to avoid overquantification or underquantification. On the other hand, when assessing activity quality, given the model’s high precision in correctly identifying tasks (ie, how a patient performs), false negatives would only reduce the amount of data analyzed, but false positives will be the primary concern, as considering these occurrences would lead to analyzing the wrong task. For example, when analyzing gait in daily living conditions, one needs to be sure that the bouts selected are gait and not another cyclic motion such as cycling, stair ascent, or shuffling. However, it is not necessary to identify all gait bouts within a day to get a relevant evaluation of a patient’s gait quality in daily living conditions [54]. Based on the precision (0.89‐0.95) and recall (0.70‐0.98) ranges observed in this study for patients with hip OA (Figure S3, Multimedia Appendix 1), precision was both higher and more stable than recall. This suggests that the model is well-suited for assessing activity quality, given its high precision in correctly identifying tasks (low number of false positives). However, its lower recall indicates reduced accuracy for assessing activity quantity, as a substantial number of task occurrences were missed (high number of false negatives).

In daily living settings, patients will be attaching IMUs themselves, leaving researchers and clinicians with limited control over sensor positioning, suggesting the need for orientation-independent models. To simulate this behavior and improve model robustness, IMU misplacements were simulated for the training dataset as a data augmentation technique by applying 180° and random-angle rotations to the axes of the raw IMU data. However, the assumption that such simulated rotations improve HAR robustness during ADL was not evaluated in this study. This would require a specific dataset with multiple trials including IMUs physically rotated, which fell outside the main scope of the study. This study addressed class imbalance by applying data augmentation techniques commonly used for time series [55]. To evaluate the effect of augmenting the training set on our results, we compared the model’s performance before and after augmentation through confusion matrices in preliminary tests (Figure S2, Multimedia Appendix 1). All classes were better recognized and improved with this method, underlying the relevance of data augmentation when analyzing time series.

### Limitations

The first limitation of this study was the low sample size and lack of external validation that limit the robustness and generalizability of the model. In other words, this study may have missed the motion abnormalities of more impaired patients (eg, patients with walking aids), which limits the applicability of the model to the general population of patients with hip OA. Indeed, the selection bias resulting from including only patients with end-stage hip OA who were scheduled for THA represents a limitation of this study. Still, providing a general model applicable to the whole population of patients with hip OA and undergoing THA was beyond the goal of this study. Moreover, the accuracy differences observed between configurations in addition to the data augmentation methods appear relevant for this population in understanding optimal IMU configurations. Nevertheless, despite the data augmentation performed to increase the data size, future work should consider training and testing the model on a larger and heterogeneous cohort of patients with hip OA, and eventually THA, to improve its applicability in clinical settings. Still, there currently seems to be no clear recommendations in the literature regarding the number of patients to include for attaining acceptable generalizability with such models. Future studies determining the number needed to make HAR models generalizable to a broader patient population would greatly contribute to the development of this field. Exploring these aspects using GRU networks is recommended since these models could achieve better performance due to their advantages in modeling temporal sequences [16]. Nevertheless, a limitation of this study is that only the Bi-GRU architecture was tested, while additional network architectures (eg, hybrid CNN-Bi-GRU models) and performance differences across model architectures could have been explored. Second, our model was trained using data collected under semistandardized conditions and not in daily living conditions. Indeed, the tour was performed outside the gait laboratory but still within the hospital, where patients were continuously filmed, and the tour remained scripted and almost identical between patients. Such protocol falls between a fully controlled environment and free-living conditions and does not represent a true patient’s performance. Therefore, the model may still produce a substantial number of false positives when encountering activities that, although belonging to the study’s defined classes, are performed differently in daily living settings due to environmental factors (eg, obstacles or weather). Still, since this study required a gold-standard identification of the tasks performed, it would thus not have been possible to perform it in full free-living conditions. Therefore, HAR models should ideally be validated with data assessed in the natural environment of patients (eg, home, work, or hobbies), despite this being very difficult to perform in practice. Third, this study did not measure patient acceptance but only assumed that the reduction of IMU number would improve wearability and patient compliance by providing a setup that is easier to install and use in daily life [56]. There was no qualitative assessment performed for patients regarding perceived ease of use and acceptance. This evaluation should be considered for future clinical applications. The fourth limitation is data augmentation: the synthetically generated data may not fully represent the data collected during ADL, thereby limiting the model’s generalizability for HAR on unseen data. However, the reported κ values resulted from evaluating the model on nonaugmented and unseen data; therefore, this did not artificially increase the resultant κ across configurations [57-59]. Nevertheless, external validation is still needed to assess real-world generalization as well as robustness to sensor misplacement. On another note, due to potential tail distortion by the Butterworth filters applied to the signals, the current solution may not be applicable for real-time applications, which presents another limitation of this study. Still, the expected use of the present application was to provide sensors to patients for recordings over multiple days and to process the data offline. Real-time applications may require different preprocessing of the signals. Another limitation is that while interoperator agreement was very high on the test subset, the full dataset was labeled by only 1 operator. Such high agreement does not eliminate the possibility of annotation bias over the entire dataset. Importantly, this study did not evaluate the psychometric measurement properties (ie, reliability, responsiveness) and interpretability of the model. Future work is encouraged to consider these psychometric properties following the COSMIN (Consensus-Based Standards for the Selection of Health Status Measurement Instruments) as it would improve the applicability of the model in clinical settings, allowing clinicians to validate algorithmic reasoning against physical observations (activities). Finally, only weight was found to be statistically different between patients and controls, with an average difference of 8.2 kg. Since higher body weight is linked to OA [60], finding controls in the same age and weight range without symptomatic OA in the lower limbs is very challenging.

### Conclusions

This work provides preliminary proof-of-concept for HAR in patients with hip OA in semistandardized conditions using a minimal setup. The findings of this proof-of-concept study suggest that, for these mobility-related activities, minimal IMU setups result in only a modest decrease in accuracy. Among the 2-IMU configurations, the 2 shanks and 2 feet achieved the highest accuracy. Single-IMU configurations achieved medium to high accuracy, with shank and trunk performing the best and pelvis and foot performing the worst. The accuracy of the HAR model was activity dependent, with gait and static sitting presenting the highest accuracy and turns and stair descent presenting the lowest. All configurations showed very high accuracy for gait detection apart from the single pelvic configuration, which still showed high accuracy. Thus, single-IMU configurations appear sufficient to recognize gait in patients with end-stage hip OA. Finally, the model’s accuracy was lower in patients than in controls, suggesting that HAR is more challenging in populations with functional impairments. To conclude, single- and 2-IMU configurations appear promising under semistandardized conditions and should be further investigated with larger and more heterogeneous hip OA cohorts.

## Supplementary material

10.2196/95116 Multimedia Appendix 1 Presenting performance metrics.

## Abbreviations

ADL: activities of daily living
Bi-GRU: bidirectional GRU
CNN: convolutional neural network
COSMIN: Consensus-Based Standards for the Selection of Health Status Measurement Instruments
GRU: gated recurrent unit
HAR: human activity recognition
IMU: inertial measurement unit
ML: machine learning
OA: osteoarthritis
THA: total hip arthroplasty
