# Supplementary material for: Minimal Sensor Configuration for Human Activity Recognition in Patients With Hip Osteoarthritis: Proof-of-Concept Study
Source: JMIR Biomed Eng. 2026 Sep 16;11:e95116. doi: 10.2196/95116 (PMC13580699; doi:10.2196/95116)
Supplement: Supplementary file 1 [file biomedeng-v11-e95116-s001.docx]

Supplementary Materials

**Figure S1 – Training and validation loss curves for the 10-fold cross validation. The solid lines represent the mean loss curves over ten folds, and the shaded curves indicate the ± standard deviation (SD)**

**Figure S2 − Confusion matrices showing model performance (8-IMU configuration) after being trained on (a) unbalanced data and (b) augmented data**

**Figure S3 − Classification metrics (precision, recall, F1-score and specificity) across IMU configurations for the patient test set (n=4)**

**Figure S4 − Classification metrics (precision, recall, F1-score and specificity) across IMU configurations for the control test set (n=3)**
